# Butanol Tolerance of *Lactiplantibacillus plantarum*: A Transcriptome Study

**DOI:** 10.3390/genes12020181

**Published:** 2021-01-27

**Authors:** Kaloyan Petrov, Alexander Arsov, Penka Petrova

**Affiliations:** 1 Institute of Chemical Engineering, Bulgarian Academy of Sciences, 1113 Sofia, Bulgaria; kaloian04@yahoo.com; 2 Institute of Microbiology, Bulgarian Academy of Sciences, 1113 Sofia, Bulgaria; alexander_arsov@abv.bg

**Keywords:** butanol, DEGs, *Lactiplantibacillus plantarum*, tolerance, transcriptomics

## Abstract

Biobutanol is a promising alternative fuel with impaired microbial production thanks to its toxicity. *Lactiplantibacillus plantarum* (*L. plantarum*) is among the few bacterial species that can naturally tolerate 3% (*v*/*v*) butanol. This study aims to identify the genetic factors involved in the butanol stress response of *L. plantarum* by comparing the differential gene expression in two strains with very different butanol tolerance: the highly resistant Ym1, and the relatively sensitive 8-1. During butanol stress, a total of 319 differentially expressed genes (DEGs) were found in Ym1, and 516 in 8-1. Fifty genes were upregulated and 54 were downregulated in both strains, revealing the common species-specific effects of butanol stress: upregulation of multidrug efflux transporters (SMR, MSF), toxin-antitoxin system, transcriptional regulators (TetR/AcrR, Crp/Fnr, and DeoR/GlpR), Hsp20, and genes involved in polysaccharide biosynthesis. Strong inhibition of the pyrimidine biosynthesis occurred in both strains. However, the strains differed greatly in DEGs responsible for the membrane transport, tryptophan synthesis, glycerol metabolism, tRNAs, and some important transcriptional regulators (Spx, LacI). Uniquely upregulated in the butanol-resistant strain Ym1 were the genes encoding GntR, GroEL, GroES, and foldase PrsA. The phosphoenolpyruvate flux and the phosphotransferase system (PTS) also appear to be major factors in butanol tolerance.

## 1. Introduction

In addition to its use as a starting reagent for chemical synthesis in various industries such as medicine and pharmacy, butanol is also the most promising alternative fuel to replace today’s gasoline. Butanol emits less unburned hydrocarbon, carbon mono- and dioxide, nitrogen oxides, and other unregulated emissions compared with conventional transport fuels [1]. It is preferred over ethanol for the spark-ignition engine because of its higher energy density, excellent combustion characteristics, less corrosiveness, lower vapour pressure and volatility, and the opportunity to be blended with gasoline at any concentration [2]. Butanol is likewise indispensable in the chemical industry for latex paints and plastics [3] and in pharmacy and cosmetics as a solvent. The butanol market is expected to reach $8.99 billion by 2026 (https://www.marketwatch.com).

Natural butanol production occurs in bacteria of genus *Clostridium* in the course of acetone–butanol–ethanol (ABE) fermentation, well-known for its low titer (less than 20 g/L), yield (typically 0.33 g/g substrate), and productivity (0.5 g·L^−1^·h^−1^) [4]. This obvious unprofitability of the process is due to the severe toxicity of butanol to bacteria [5,6]. The effects of butanol on microbial cells include the destruction of the phospholipid bilayer and the membrane lipopolysaccharides [7], inhibition of the cell membrane ATPase activity, and the glucose absorption [8]. This is why several genetic approaches have been applied to enhance microbial resistance to butanol; that is, chemical mutagenesis [9,10,11], genomic libraries construction and screening [12,13], forced genomic evolution [14,15], and genome shuffling [16]. Transcriptomics is another valuable tool to study the molecular mechanisms of the butanol stress response [17]. The crucial role of *groESL*, *dnaK, hsp90*, *hsp18*, *clpC*, and *htrA* overexpression for butanol tolerance of *Clostridium acetobutylicum* has been confirmed by DNA microarray-based transcriptional analysis [18,19]. Transcriptomic analysis has shown that *groEL* overexpression increases the butanol resistance of *Escherichia coli*, *Bacillus subtilis*, *Lactococcus lactis*, and *Lacticaseibacillus paracasei* NFBC 338 [20,21]. Transcriptomic methods have also revealed the importance for butanol tolerance of efflux pump components, such as those encoded by *acrA* and *acrB* in *E. coli* [16] or *srpB* in *Pseudomonas putida* [22], the two-component system encoded by *btrTM* [23], the master regulator Spo0A in *C. acetobutylicum* [8], and the *nrps3* biosynthetic cluster in *C. saccharoperbutylacetonicum* N1–4 [24].

Natural butanol-resistant species can be a valuable genetic reservoir for improving the weak tolerance of the natural butanol producers, or they may serve as a heterologous host of the clostridial ABE pathway and its synthetic analogues [4]. In some cases, such attempts resulted in negligible butanol production, for instance, by *Ps. putida* [25], *Levilactobacillus brevis* [26], *B. subtilis* [25], *Lc. lactis* [27], *Saccharomyces cerevisiae* [28,29], and *Methylobacterium extorquens* [30]. However, some of the engineered strains such as *E. coli* EB243 are superior in the production of butanol and limited in the synthesis of by-products [31].

*L. plantarum* is a species that participates in lactic acid fermentation of numerous beverages and foods, including wine, yoghurt, boza, sourdough, sauerkraut, and sausages [32,33,34], and is typically resistant to environmental stress caused by acidic pH, extremes in temperature, osmotic pressure, oxygen, starvation, and high alcohol concentrations [35,36]. Selected strains of *L. plantarum* can grow at butanol concentrations up to 3–4% (*v*/*v*) [34,37]. Despite these promising data, the reasons for the butanol resistance of *L. plantarum* have been studied very little on a genetic level and remain elusive [38].

The present study aims to reveal the genetic foundations of the natural butanol tolerance of *L. plantarum* using comparative transcriptomics. For the first time, differential gene expression is studied in two strains of the same species, but with different ability to withstand butanol stress, the highly tolerant Ym1, and the relatively sensitive 8-1. By the juxtaposition of gene expression patterns, the study proposes to elucidate the following: (1) the species-specific determinants of the natural butanol resistance of *L. plantarum* and (2) the strain-specific differences between butanol-resistant and butanol-sensitive members of the same species.

## 2. Materials and Methods

### 2.1. Bacterial Strains, Media, and Butanol Tolerance Assay

*L. plantarum* strains Ym1 and 8-1 were previously isolated from Bulgarian yoghurt and Boza and identified by 16S rRNA gene sequencing (accession no. MH430035 and KU513396). The strains were cultivated in 200 mL MRS medium (Merck KGaA, Darmstadt, Germany), inoculated with 1% overnight culture (OD ~ 2.0), and grown at 37 °C, in 250-mL borosilicate glass bottles (ISOLAB Laborgeräte GmbH, Eschau, Germany), stirring at 100 rpm on rotary shaker.

For estimation of strain’s butanol resistance degree, the relative growth rate in MRS medium, supplemented with 1% to 3% (*v*/*v*) n-butanol (Sigma-Aldrich Chemie GmbH, Germany) was compared with the control (MRS without solvent). Natural logarithm (ln) of the average optical densities of the cultures obtained during three to eight independent experiments was calculated. The relative growth rates were estimated as a ratio between the specific growth rates in the presence and absence of butanol.

### 2.2. Analytical Methods

The cell growth was monitored by measuring the OD of the culture at a wavelength of 600 nm using UV/VIS Spectrophotometer (Thermo ScientificTM).

The concentrations of glucose, lactic acid, and butanol were determined by HPLC using HPLC column Aminex HPX-87H (BioRad Laboratories, Hercules, CA, USA) at 65 °C, with a mobile phase 5 mM sulfuric acid at a flow rate of 0.6 mL/min, and an RI detector (PerkinElmer series 10, PerkinElmer, Waltham, MA, USA).

RNA concentrations and purity (Abs260/Abs280 ratio) were determined using Quawell UV Spectrophotometer Q3000 (Quawell Technology Inc., San Jose, CA, USA).

### 2.3. RNA Isolation, Libraries Construction and Sequencing

Total RNA was isolated with GENE Matrix Universal RNA Purification Kit (EURx, Gdańsk, Poland) from cultures grown for 8 h in MRS with and without 2% (*v*/*v*) butanol, according to the manufacturer’s instructions.

Libraries’ construction and sequencing were done by Macrogen Inc., Seoul, Korea. RNA-seq libraries were prepared from 1 µg of total RNA using TruSeq RNA sample preparation kit (Illumina, San Diego, CA, USA). RiboZero rRNA removal kit was used for rRNA removal; cDNA synthesis and adaptors addition were according to Illumina protocol. After libraries’ quantification, 100 bp paired-end sequencing was done on Illumina Novaseq 6000. Quality check on the raw sequences was done using program FastQC v0.11.7 (http://www.bioinformatics.babraham.ac.uk/projects/fastqc/).

Then, the assays were analysed by HTseq version 0.10.0 (http://www-huber.embl.de/users/anders/HTSeq/doc/overview.html).

Raw transcriptome sequencing data were submitted to NCBI with the accession numbers PRJNA675296 (*L. plantarum* Ym1) and PRJNA675298 (*L. plantarum* 8-1).

### 2.4. Bioinformatics Analysis

The raw paired-end reads were trimmed and quality controlled using Trimmomatic v.0.38 (http://www.usadellab.org/cms/?page=trimmomatic) with a sliding window set to length 4 and Phred quality score 15, and minimum length to 75. Trimmed reads (Supplementary Table S1) are mapped to the reference genome with Bowtie 1.1.2 (http://bowtie-bio.sourceforge.net/index.shtml). The clean reads were separately aligned to the reference genome of *L. plantarum* WCFS1, NCBI GenBank №AL935263.

Transcriptome analyses were successfully performed on four paired-ends samples (Ym1–2%, Ym1-control, 8-1–2%, 8-1-control). To identify differentially expressed genes (DEGs), the expression levels of the transcripts were calculated using the fragments per kilobase of reading per million mapped reads (RPKM) method. The expression profile was calculated for each sample and gene as a reading count. The fold change (FC), ExactTest (using edgeR), and hierarchical clustering were used as statistical methods. The significant results are selected on conditions of |FC| ≥ 2 & exactTest raw *p*-value < 0.05. The results showed 656 genes, which satisfied this condition in at least one of comparison pairs. Boxplot of expression difference between samples is presented in Supplementary Figure S1.

## 3. Results and Discussion

### 3.1. Selection of Butanol-Tolerant and Butanol-Sensitive Strains

**Figure 1:**
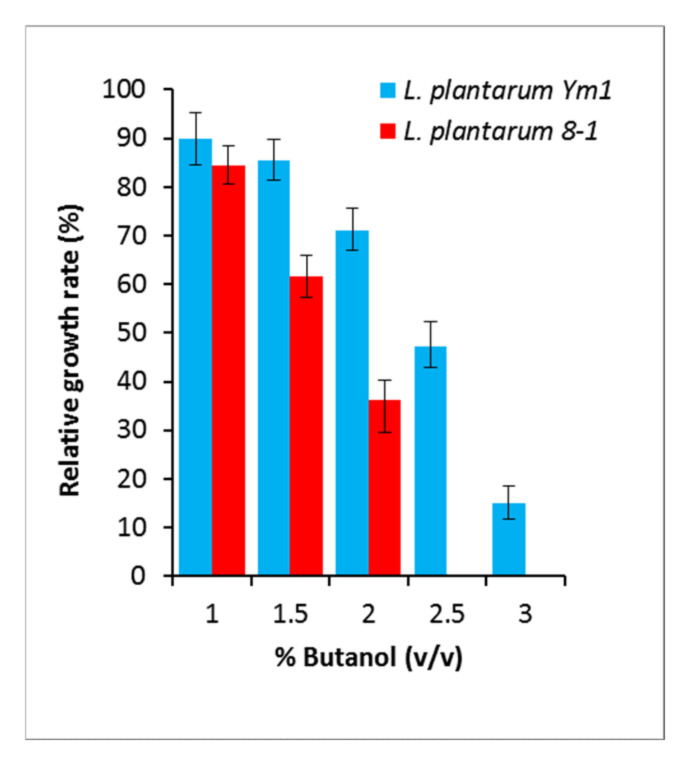
Relative growth rates of *L. plantarum* Ym1 and *L. plantarum* 8-1 in MRS medium supplemented with 1%–3% (*v*/*v*) butanol. Growth in MRS without butanol is considered as 100%. The presented standard deviations are obtained from at least three different experiments.

The strains Ym1 and 8-1 were selected as the most resistant and the most sensitive to butanol among ten *L. plantarum* strains possessing the same morphological, biochemical, and growth characteristics. Under optimal growth conditions, the butanol-tolerant strain Ym1 showed the following relative growth rates (RGR): 71.1% in medium containing 2.0% (*v*/*v*) butanol, 47.3% at 2.5% (*v*/*v*), and 14.9% at 3.0% (*v*/*v*). Conversely, the butanol-sensitive strain 8-1 did not show any growth at 2.5% (*v*/*v*) butanol, and reached only 36.3% RGR at 2.0% (*v*/*v*) (Figure 1). Therefore, for the analysis of differential gene expression during butanol challenge, a concentration of 2.0% (*v*/*v*) butanol was chosen.

**Figure 2:**
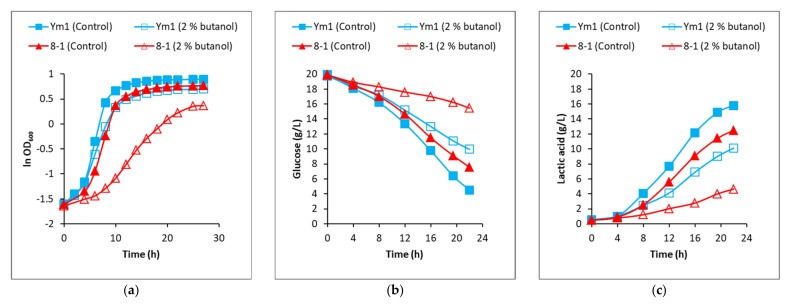
Growth kinetics of *L. plantarum* strains Ym1 and 8-1 exposed to butanol stress. (**a**) Biomass formation of *L. plantarum* Ym1 and *L. plantarum* 8-1 in control MRS medium and MRS supplemented with 2% (*v*/*v*) butanol. Natural logarithm (ln) of the average optical density of the cultures from three different experiments is presented. (**b**) Time profile of glucose consumption. (**c**) Time profile of lactic acid formation.

The growth kinetics of the strains Ym1 and 8-1 cultured in MRS containing 2% (*v*/*v*) butanol and in MRS without butanol (controls) are presented in Figure 2a. Substrate consumption and lactic acid (LA) production kinetics in Figure 2b,c revealed that, notwithstanding the butanol presence, *L. plantarum* strains Ym1 and 8-1 performed homofermentative conversion of glucose to LA, with a yield close to the theoretical maximum (0.96–0.99 g LA per g consumed glucose). However, when the strains were subjected to butanol stress, glucose consumption decreased. For DEGs’ analyses, total RNAs were extracted using 8 h cultures, the time point corresponding to the linear range of exponential growth of the controls (Figure 2a).

### 3.2. Overview of Differentially Expressed Genes (DEGs)

To examine the different gene expression profiles, whole transcriptome sequencing and annotation based on gene ontology pathway information for the reference *L. plantarum* WCFS1 genome (NC_004567.2) was performed. Two pairs of samples were analysed and compared: (1) Ym1 grown in MRS containing 2% (*v*/*v*) butanol, to Ym1 grown in MRS (control); and (2) 8-1 grown in MRS with 2% (*v*/*v*) butanol, to 8-1 grown in MRS (control). From 3115 genes analysed, 658 genes were excluded, because they gave at least one zero count out of the four samples. The other 2457 genes were used for the statistical analysis. A total of 656 DEGs were found, upregulated 156 against 271 and downregulated 163 against 245 in Ym1 and 8-1, respectively (Supplementary Figure S2). The genes overexpressed in Ym1, but downregulated in 8-1, and vice-versa, are listed in Table 1.

**Figure 3:**
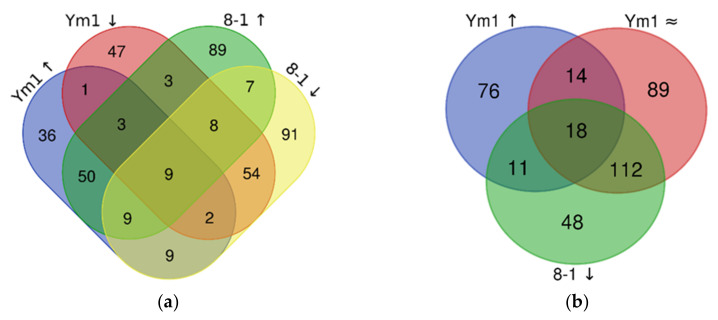
Venn diagrams of DEGs (differentially expressed genes) in *L. plantarum* strains Ym1 and 8-1 under butanol stress. (**a**) Total number of DEGs in both strains; (**b**) DEGs downregulated in 8-1, overexpressed in Ym1, and genes without significant change in expression in Ym1 (±2-fold change). Symbols: ↑, overexpressed genes; ↓, downregulated genes; ≈, genes without significant change in expression.

Venn diagrams showed that Ym1 and 8-1 share 50 overexpressed and 54 downregulated genes (Figure 3a), thus indicating the species-specific determinants of the butanol resistance in *L. plantarum*.

**Figure 4:**
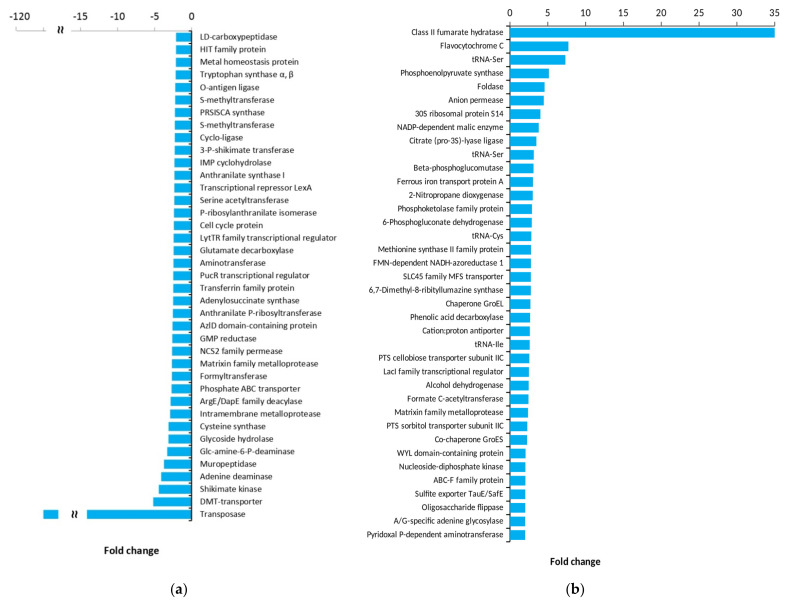
Unique DEGs in *L. plantarum* strain Ym1. (**a**) Downregulated genes; (**b**) upregulated genes.

Notably, some of the enzymes are encoded by several gene copies (Figure 3b) with a different fold change (FC). Thirty-eight unique genes in the butanol-tolerant Ym1 were overexpressed and 39 were downregulated, as DEGs with FC more than two-fold are shown in Figure 4. DEGs with the largest deviations in FC values in both strains are presented in Supplementary Figure S3.

### 3.3. Cell Envelope and Cell Membrane Alterations

Butanol stress alters first of all the content and structure of the cell envelope. The results from the differential gene expression showed that *L. plantarum* most probably increases the production of exopolysaccharides (EPS) to build up a physical barrier between the solvent and the cell wall, as well as to increase the cell envelope hydrophilicity, which has been shown to enhance butanol resistance [34]. Six DEGs with FC between +2.0 and +3.4 responsible for EPS synthesis are overexpressed only in Ym1: *wzx* gene encoding oligosaccharide flippase (lp_2099), *wzy* for polysaccharide polymerase (lp_2101), and three genes encoding different ‘polysaccharide biosynthesis’ proteins (lp_2108, lp_1221, and lp_1225), well-characterized as part of the *cps* gene clusters in *L. plantarum* WCFS1 [39].

**Figure 5:**
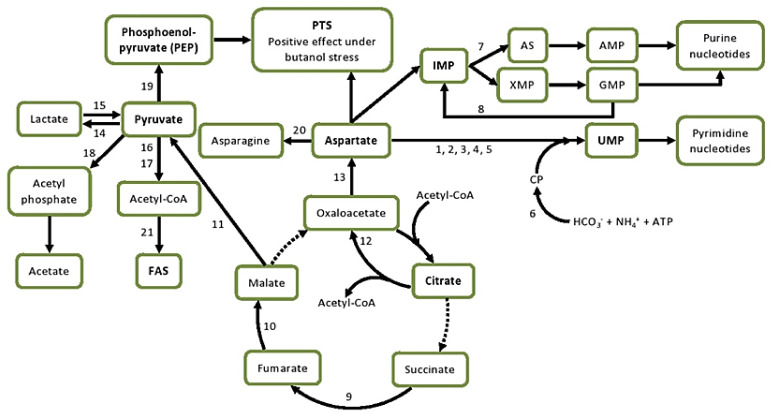
Metabolic redistribution of citrate, aspartate, and pyruvate under butanol stress in *L. plantarum* strains 8-1 and Ym1. Regulated enzymes as explained in the text: 1, aspartate carbamoyltransferase; 2, dihydroorotase; 3, dihydroorotate dehydrogenase; 4, orotate phosphoribosyltransferase; 5, orotidine-5’-phosphate decarboxylase; 6, carbamoyl phosphate synthase; 7, adenylosuccinate synthase; 8, guanosine monophosphate reductase; 9, succinate dehydrogenase; 10, fumarate hydratase; 11, NAD(P)-dependent malic enzyme; 12, citrate lyase; 13, aminotransferase; 14, L-lactate dehydrogenase; 15, L-lactate oxidase; 16, pyruvate dehydrogenase complex (PDHC); 17, pyruvate formate lyase; 18, pyruvate oxidase; 19, phosphoenolpyruvate synthase 20, asparagine synthase; 21, acetyl-CoA carboxylase. Abbreviations: AMP, adenosine monophosphate; AS, adenylosuccinate; ATP, adenosine triphosphate; FAS, fatty acid synthesis; GMP, guanosine monophosphate; IMP, inosine monophosphate; PEP, phosphoenolpyruvate; PTS, PEP: carbohydrate phosphotransferase system; UMP, uridine monophosphate; XMP, xanthosine monophosphate. Continuous lines denote existing metabolic pathways; dotted lines denote metabolic pathways, which appear non-functional according to our transcriptomic data for both strains.

The genes involved in fatty acid synthesis (FAS) *fabD* (encoding Acyl-carrier protein-S-malonyltransferase), *fabF* (for 3-oxoacyl-ACP synthase), *fabG* (3-oxoacyl-ACP reductase), *fabZ* (3R-hydroxymyristoyl-ACP dehydratase), and *fabI* (for enoyl-acyl-carrier protein) remained without a change in expression during butanol stress in the resistant strain Ym1, whereas they all were downregulated in 8-1, an effect similar to the one observed in *L. plantarum* strains WCSF1 and Y44 exposed to ethanol and oxidative stress [40,41]. Several subunits of the enzyme acetyl-CoA carboxylase, responsible for the first step of FAS (the carboxylation of acetyl-CoA to malonyl-CoA), are between three- and fourfold downregulated in the butanol-sensitive 8-1 (Supplementary Figure S3). Downregulation of the E1 component of pyruvate dehydrogenase complex (PDHC), together with significant (11-fold) downregulation of an activating protein for the pyruvate formate lyase complex, will further decrease the level of acetyl-CoA, the major raw material of FAS (Figure 5). In contrast, the same genes appear unaffected in Ym1.

We found five genes encoding WxL domain-containing proteins in Ym1, and four in 8-1. One of them, for WP011102288.1, is significantly upregulated (+12- and +14-fold) in both strains (Supplementary Figure S3). The WxL domain is responsible for peptidoglycan binding and was first described as C-terminal domain in cell-surface proteins in *L. plantarum* [42]. In *Enterococcus faecium*, it is involved in extracellular interactions that may contribute to colonization and virulence [43].

### 3.4. Membrane Transport

The most significant effect of butanol stress is the disturbance of sugar uptake, which results in a decrease in the intracellular ATP levels and cell starvation. In *L. plantarum*, the transmembrane sugar transport is mainly provided by the phosphotransferase system (PTS), an active transport supported by phosphoenolpyruvate (PEP) as an energy source (Figure 5). PTS transporters exhibit a striking pattern of differential expression under butanol stress in *L. plantarum* (Table 2). Our data show 23 DEGs related to PTS transporters, half of them concerned with the membrane-bound subunit C. From those upregulated in 8-1, lp_2531 (FC +3.22) appears to be specific for N-acetylglucosamine and may play a role in the recycling of peptidoglycan. All 19 genes are downregulated in 8-1, including some encoding PTS transporters for mannitol and cellobiose (11- to 17-fold), to a lesser extent also for sorbitol (3.11- to 5.28-fold) and glucose (2.3-fold), thus indicating severely impaired sugar uptake. In stark contrast, only eight genes are affected in Ym1, and only four of them are downregulated. Another notable difference concerns all three subunits (A, B, and C) of a mannitol transporter. This is the only case among the PTS transporters, which are regulated in opposite directions: 3.22-fold downregulated in 8-1 and 6.48-fold upregulated in Ym1 (lp_0230). The only transporter upregulated in both strains, 2.48-fold in 8-1 and 4.35-fold in Ym1, is fructose-specific. Two genes for transporters (lp_3654 and lp_0286, *celB*) are uniquely upregulated in Ym1, the first for sorbitol (2.29-fold) and the second for cellobiose (2.55-fold). The sugar uptake in Ym1 under butanol stress is much less affected compared with the more sensitive strain 8-1.

We observed the strongest downregulation among the PTS transporters in two C-subunits: lp_0265 and lp_0264. They were 14.69- and 25.45-fold downregulated in 8-1 and 18.57- and 38.68-fold in Ym1, respectively. Based on a 73.2% match with the respective proteins in *Listeria monocytogenes*, they seem to be involved in trehalose transport. This agrees with the pronounced downregulation of lp_0263, encoding α, α-phosphotrehalase, which hydrolyses trehalose-6-phosphate to glucose and glucose-6-phosphate (with decreases of 19-fold in 8-1 and 30-fold in Ym1). However, it does not agree with the downregulation of the trehalose (*tre*) operon repressor *treR* with FC (−11) in 8-1 and (−23) in Ym1. These data are controversial, but suggestive. Trehalose and the *tre* operon have been implicated in cryoprotection in *L. acidophilus* [44] as well as in stress response and toxin production in *Streptococcus mutans* [45]. Their role in butanol stress needs to be explored further.

In Gram-negative bacteria, butanol tolerance is primarily associated with reverse transport of the solvent by energy-dependent efflux pumps [46], with probable analogues in Gram-positive bacteria [47]. DEGs in the analysed *L. plantarum* strains showed the presence of multidrug efflux transporters that are probably involved in butanol efflux: SMR (lp_3285), about twelve members of the MFS and DHA2 families, and an MMPL family transporter (lp_0295). All multidrug transporters were found to be from two- to sixfold overexpressed in both strains, but the last one is uniquely overexpressed in Ym1.

### 3.5. Chaperones and Chaperonins Expression

DEGs encoding co-chaperone GroES (+2.70 FC) and chaperonin GroEL (+2.26 FC) are uniquely overexpressed in the butanol-resistant strain Ym1. Thus, our results confirm their importance for the butanol stress response, as has been shown for *B. subtilis*, *E. coli*, *Lc. lactis*, *L. paracasei*, and *C. acetobutylicum* [19,20,21]. Besides, being responsible for protein folding, GroES and GroEL are involved in the core stress response to ethanol in *L. plantarum* [40]. Another gene overexpressed only in Ym1 is *prsA*, encoding extracellular foldase (lp_3193), an ubiquitous glycoprotein anchored to the outer layer of the cell membrane [48]. PrsA is responsible for the folding and maturation of many secreted proteins and is known to contribute to protein folding and secretion in *Lacticaseibacillus rhamnosus* GG [49]. We found that the foldase gene is 4.6-fold overexpressed in Ym1, but remains unaltered in 8-1.

### 3.6. Pyruvate Metabolism

Our transcriptomic data suggest an increased production of pyruvate under butanol stress in *L. plantarum*, which may well lead to increased levels of PEP and, therefore, better functioning of the PTS transporters. The key genes and enzymes are the following: *spxB*, encoding pyruvate oxidase; *ppsA*, encoding phosphoenolpyruvate synthase (PEPS); and *pck* for phosphoenolpyruvate carboxykinase (Figure 5). The gene lp_1912 encoding enzyme PEPS (EC 2.7.9.2), which is responsible for the conversion of pyruvate to phosphoenolpyruvate (PEP), is fivefold upregulated in Ym1, but unchanged in 8-1. Because PEP is the sole energy source of the PTS transporters, its production may correlate with changes in that system.

As discussed above, PTS transporters are much more strongly downregulated in 8-1 under butanol stress. Hence, the PEP obtained by the *ppsA* overexpression in Ym1 is likely to be used in this direction rather than, for example, gluconeogenesis. Lactate production kinetics revealed that, notwithstanding the butanol stress, almost all consumed glucose was converted to lactate (Figure 2c). In strain 8-1, two genes for pyruvate oxidase, *poxB* and *spxB*, are 4-fold upregulated and 11-fold downregulated, respectively, while another one for lactate oxidase is 4-fold upregulated. Moreover, *pdhA* and *pdhB* encoding the α and β subunits of E1 component of the pyruvate dehydrogenase complex (PDHC) are severely downregulated in 8-1 (14- and 17-fold, respectively), thus suggesting that pyruvate is primarily directed to PEP and lactate. In the butanol-resistant Ym1, lactate oxidase and the PDHC are largely unaffected by butanol stress, but the pyruvate oxidase is nearly fivefold downregulated. Lactate dehydrogenase (LDH) is nearly fivefold upregulated (against only twofold in 8-1), which is in agreement with the homofermentative process of glucose conversion. An additional source of pyruvate may be the NAD(P)-dependent malic enzyme, which dehydrogenates and decarboxylates malate to pyruvate. The responsible gene *mae* (lp_1105) is upregulated in both strains, but more strongly in Ym1 (3.8-fold) than in 8-1 (2.6-fold). Another possible application of pyruvate lies in its contribution to metabolic reprogramming, as described in *Ps. fluorescens* [50], and this effect needs future consideration.

### 3.7. Nucleotide Metabolism

**Figure 6:**
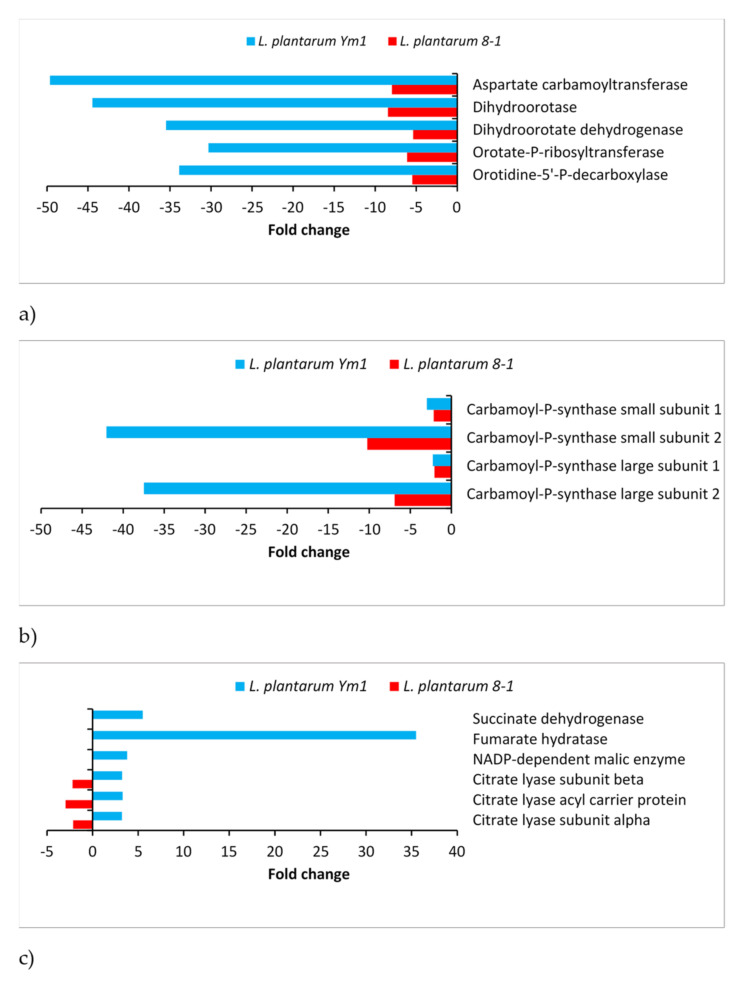
Differentially expressed genes (DEGs) involved in redistribution of citrate, aspartate, and pyruvate in *L. plantarum* strains Ym1 and 8-1 during butanol stress. (**a**) Enzymes involved in the biosynthesis of pyrimidine nucleotides. (**b**) DEGs encoding carbamoyl phosphate synthase: lp_0527 and lp_0526, small subunit 1 and 2; lp_0526 and lp_2700 (*carB*), large subunit 1 and 2. (**c**) Enzymes involved in the tricarboxylic acid (TCA)-aspartate shuttle.

Inhibition of the pyrimidine biosynthesis was observed in *L. plantarum* Y44 under oxidative stress [41]. We found a similar effect in *L. plantarum*, pronounced in 8-1, but far stronger in Ym1. The first five enzymes from the biosynthetic pathway of uridylate (UMP) are 5- to 8-fold downregulated in 8-1 and 30- to 50-fold downregulated in Ym1 (Figure 6a). This is a striking difference, certainly relevant to Ym1’s greater butanol resistance. The effect is mirrored in the downregulation of both subunits of the carbamoyl phosphate synthase (7- to 10-fold in 8-1, 37- to 42-fold in Ym1), the enzyme responsible for the formation of carbamoyl phosphate (Figure 6b), one of the major substrates for the biosynthesis of pyrimidine nucleotides, together with aspartate, whose intracellular accumulation has been shown to protect *Lacticaseibacillus casei* from acid stress [51].

Aspartate accumulation is a probable consequence of the inhibition of the pyrimidine biosynthesis in *L. plantarum* under butanol stress. This is further supported by the downregulation of asparagine synthase, an enzyme that catalyses the conversion of aspartate into asparagine, 5.4- and 3.7-fold downregulated in Ym1 and 8-1, respectively. Another possible route that may lead to increased levels of aspartate is through intermediates from the TCA (tricarboxylic acid) cycle (Figure 6c). The most highly upregulated gene in Ym1 (35.5-fold) is the one for fumarate hydratase (lp_1112). Following other upregulated genes, such as those for an FAD-dependent oxidoreductase with succinate dehydrogenase activity (lp_3125), NADP-malic enzyme (lp_1118), and citrate lyase (lp_1109, *citF*), we propose the existence of a TCA-aspartate shuttle in which intermediate metabolites from the TCA cycle, notably citrate and oxaloacetate, may contribute to increased levels of aspartate in Ym1 (Figure 5).

At least one aminotransferase gene is uniquely twofold upregulated in Ym1 (lp_2888), though it is possible that others may also contribute to the amination of oxaloacetate to aspartate. The purine biosynthesis is much less affected in both strains, but there is a significant difference regarding inosinate (IMP), the precursor of both adenylate (AMP) and guanylate (GMP). Adenylosuccinate synthase (lp_3270), a GTP-dependent enzyme that uses IMP and aspartate as substrates, is 2.5-fold downregulated in Ym1, but unchanged in 8-1 under butanol stress. The same effect is even more pronounced concerning the GMP reductase gene (lp_3271). This enzyme is responsible for the oxidative deamination of GMP to IMP. It is 2.6-fold downregulated in Ym1 and 2-fold upregulated in 8-1. The net effect of this, in Ym1 as opposed to 8-1, would be reduced production of IMP and AMP, and correspondingly saving aspartate for alternative pathways possibly related to increased butanol tolerance.

Summarized, our data suggest that aspartate is likely to play an important role in the increased butanol tolerance of *L. plantarum* Ym1, although the mechanism remains to be explored. The role of aspartate in butanol resistance may be a fruitful subject for future research.

### 3.8. Glycerol Metabolism

**Figure 7:**
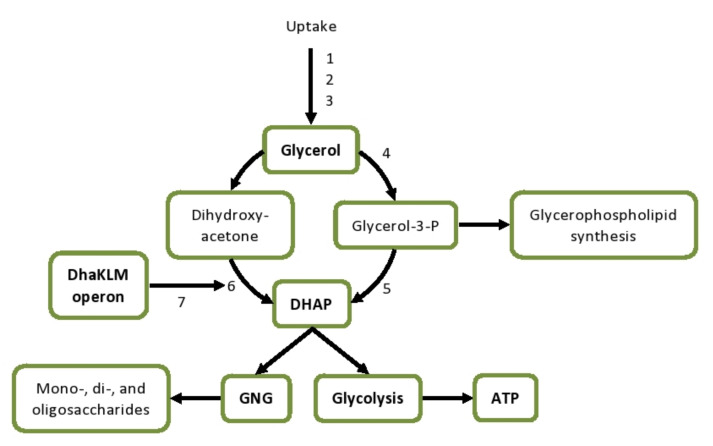
Glycerol uptake and metabolism in *L. plantarum* Ym1 and 8-1 under butanol stress. DEGs encode the following enzymes, transport proteins, and transcription factors: 1–3, uptake facilitator proteins; 4, glycerol kinase; 5, glycerol 3-phosphate oxidase; 6, PTS-dependent DHA kinase; 7, DhaQ coactivator of the *DhaKLM* operon. Abbreviations: DEGs, differentially expressed genes, ATP, adenosine triphosphate; DHA, dihydroxyacetone; DHAP, dihydroxyacetone phosphate; GNG, gluconeogenesis.

**Figure 8:**
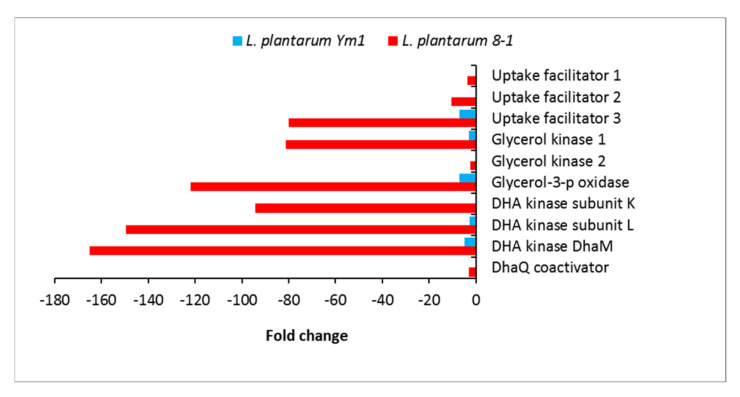
Differentially expressed genes (DEGs) involved in glycerol uptake and conversion in *L. plantarum* strains Ym1 and 8-1 during butanol stress. Lp_0108, lp_0372, and lp_0171 encode glycerol uptake facilitator proteins GlpF 1, 2, and 3, respectively; lp_0370 and lp_0834 (*glpK*) encode glycerol kinase 1 and 2, respectively.

One of the strongest effects on a transcriptional level is the inhibition of the glycerol uptake and metabolism in *L. plantarum* 8-1 (Figure 7). Three genes (lp_0108, lp_0372, lp_0171) encoding glycerol uptake facilitator proteins GlpF are downregulated between 3- and 80-fold. Lp_0370 encoding glycerol kinase GlpK, the enzyme responsible for the production of glycerol-3-phosphate, is also 80-fold downregulated (Figure 8). This indicates strong suppression of the phosphorylation pathway of glycerol utilization. As glycerol-3-phosphate is a major substrate for the biosynthesis of glycerophospholipids (Figure 7), this is one way in which butanol stress is likely to affect the composition and fluidity of the cell membrane. Theoretically, glycerol-3-phosphate may be regenerated via isomerisation and hydrogenation of glyceraldehyde-3-phosphate, but this is extremely unlikely. Glyceraldehyde-3-phosphate is produced midway through the glycolysis, after the two ATP-consuming steps, but before the two ATP-producing steps; hence, its diversion into anabolic direction is most likely to disrupt the cell’s energy balance.

The glycerol metabolism is closely related to that of dihydroxyacetone (DHA) and dihydroxyacetone phosphate (DHAP). Here, we observe another set of interlinked effects that may have far-reaching consequences for the cell metabolism. Glycerol-3-phosphate oxidase (GlpO), an enzyme producing DHAP directly, shows 122-fold downregulation in the strain 8-1. As DHAP is part of the glycolysis, this downregulation effectively prevents using glycerol as an energy source. DHAP can also be produced by phosphorylation of DHA by the DhaKL kinase, which uses PEP as a source of phosphate. Remarkably, the K and L subunits of DhaKL are 94- and 149-fold downregulated, respectively, in the strain 8-1. This is further confirmed by massive downregulation (165-fold) of the PTS-dependent phosphotransferase subunit DhaM. As *L. plantarum* does not possess glycerol dehydrogenase [52] and cannot convert glycerol directly to DHA, this pathway of glycerol dissimilation is blocked at the start.

On the whole, our transcriptomic data strongly support the hypothesis that the suppression of glycerol metabolism is one of the major negative effects of butanol stress. *L. plantarum* is a well-documented producer of glycerol [53], but butanol-sensitive strains such as 8-1 may not be able to utilize it in the presence of butanol. Some of the glycerol-related genes mentioned above, for instance, one of the uptake facilitators and the glycerol-3-phosphate oxidase, are downregulated in Ym1 as well, but to a much lesser degree (Figure 8).

### 3.9. Amino Acid Metabolism

**Figure 9:**
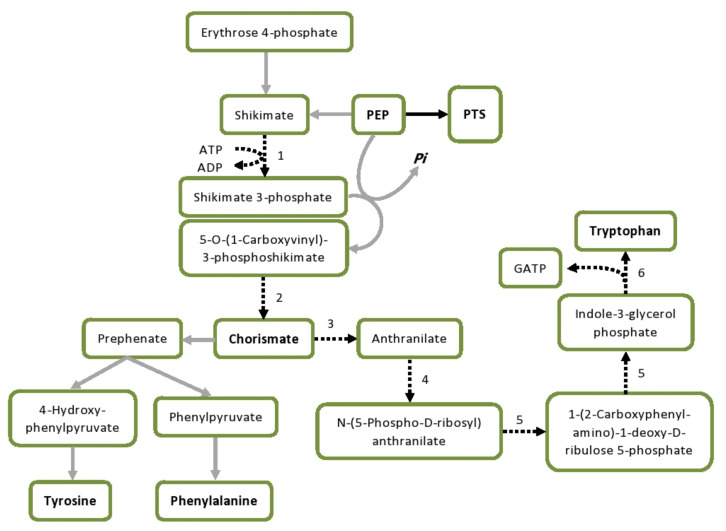
Inhibition of tryptophan biosynthesis and redistribution of PEP under butanol stress in *L. plantarum* Ym1. Downregulated steps are marked with dotted lines. Enzymes: 1, shikimate kinase; 2, chorismate synthase; 3, anthranilate synthase; 4, anthranilate phosphoribosyltransferase; 5, phosphoribosyl anthranilate isomerase; 6, tryptophan synthase. Abbreviations: GATP, glyceraldehyde 3-phosphate; GNG, gluconeogenesis; PEP, phosphoenolpyruvate; Pi, inorganic phosphate; PTS, PEP: carbohydrate phosphotransferase system.

Aromatic amino acids have been implicated in the stress response to various stimuli in different species. Their biosynthesis (shown in Figure 9) is inhibited in *Ps. putida* BIRD-1 in the presence of 0.3% butanol [54]. Tyrosine is a major hub metabolite in *Clostridium* under butanol stress [55] and its decarboxylation is one of the more unusual transcriptional responses in *L. brevis* subjected to 2% (*v*/*v*) butanol [56]. Recently, transcriptional upregulation of genes involved in the biosynthesis of aromatic amino acids was observed in *L. plantarum* under AAPH (2,2-azobis(2-methylpropionamidine) dihydrochloride) stress [41]. Our data suggest strongly inhibited synthesis of aromatic amino acids during butanol stress in Ym1. Chorismate synthase, the enzyme responsible for one of the major precursors of aromatic amino acids, is 2-fold downregulated in Ym1, but 2.6-fold upregulated in 8-1. The minor role of aromatic amino acids in Ym1 is further confirmed by the downregulation of shikimate kinase (4.4-fold), a key enzyme in the production of chorismate, and concerted suppression of the tryptophan biosynthesis. All enzymes involved in the process are consistently downregulated between 2.11- and 3.25-fold, including both α and β subunits of the tryptophan synthase (Table 3). This is in contrast with butanol stress in *E. coli*, in which tryptophan synthesis is upregulated [57], a timely reminder that many facets of the stress response are species-specific. One possible reason for *L. plantarum* Ym1 to shut down the tryptophan synthesis may be to save PEP for the PTS transporters, as in the case of pyruvate discussed above. It is interesting to note that component II of the anthranilate synthase is also downregulated in *L. plantarum* strain 8-1. In combination with the increased levels of chorismate synthase, this seems to suggest that chorismate is diverted into the synthesis of tyrosine, phenylalanine, and possibly other important metabolic precursors.

### 3.10. Translation

**Figure 10:**
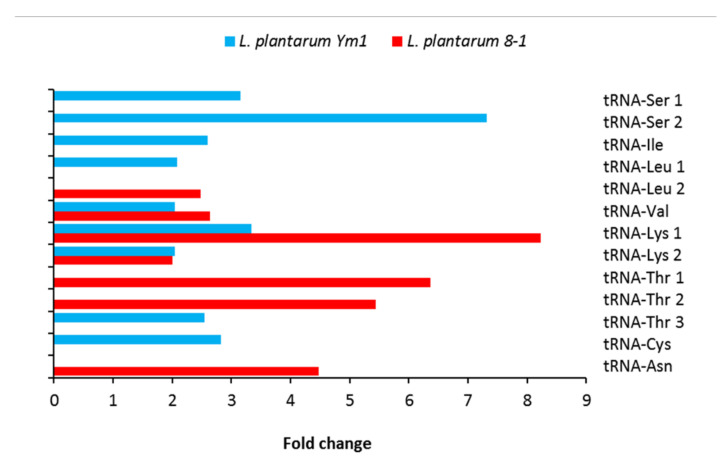
Differentially expressed genes (DEGs) in *L. plantarum* strains Ym1 and 8-1 during butanol stress encoding transport RNAs. Gene and tRNA: lp_tRNA50, tRNA-Ser 1; lp_tRNA25, tRNA-Ser 2; lp_tRNA51, tRNA-Ile; lp_tRNA34, tRNA-Leu 1; lp_tRNA14, tRNA-Leu 2; lp_tRNA27, tRNA-Val; lp_tRNA01, tRNA-Lys 1; lp_tRNA58, tRNA-Lys 2; lp_tRNA69, tRNA-Thr 1; lp_tRNA05, tRNA-Thr 2; lp_tRNA57, tRNA-Thr 3; lp_tRNA33, tRNA-Cys; lp_tRNA13, tRNA-Asn.

We found thirteen different tRNAs differentially expressed under butanol stress (Figure 10). The most significant difference between the two strains is concerned with tRNA-Ser. Two of them are 3.15- and 7.32-fold upregulated in Ym1, but apparently unaffected in 8-1.

The serine-rich repeat proteins (SRRPs) are common on the surface of various lactobacilli such as *Limosilactobacillus reuteri*, but none of them have been found in the genome of *L. plantarum* [58]. Further studies will be needed to explain this curious pattern of tRNA-Ser expression in *L. plantarum* Ym1 under butanol stress. Somewhat paradoxically, the serine-tRNA ligase is downregulated in Ym1, though only 2.22-fold, as opposed to 12.48-fold in 8-1. A similar effect is observed with tRNA-Thr and lp_1514 (*thrS*) encoding threonine-tRNA ligase. There is a difference, however. Three different tRNA-Thr are upregulated, two in 8-1 (6.36- and 5.44-fold) and one in Ym1 (2.54-fold), but the threonine-tRNA ligase is downregulated only in 8-1 (2.26-fold). Serine/threonine-rich proteins are known to promote aggregation in *L. plantarum* NCIMB 8826 [59], but we found none among the DEGs in our two strains. We did, however, fine other differentially expressed tRNAs, sometimes in both strains (valine, lysine), sometimes only in Ym1 (cysteine, isoleucine). Besides, one 30S ribosomal protein (S14) is 4-fold upregulated in Ym1 and two 50S ribosomal proteins, L29 and L34, are 2.4- and 2.2-fold downregulated, respectively, in 8-1, further suggesting more active translation in Ym1 under butanol stress.

### 3.11. Transcription Factors and Regulators

We found a total of 44 differentially expressed transcription factors. Although the percentage of upregulated is similar in both strains (42 in 8-1, 34 in Ym1), the number is considerably larger in 8-1 (35) than in Ym1 (19). Only 12 transcription factors are regulated in both strains (Table 4). The only one regulated in opposite directions—2.83-fold upregulated in Ym1, 7.83-fold downregulated in 8-1—belongs to the GntR family, a large group of transcription factors associated, among other things, with various types of stress in different species of LAB [60,61]. Two other members of the same family are 3.5- to 3.6-fold downregulated in 8-1. One of them is identified as a repressor of the trehalose operon based on an 87% identity with the gene of *Pseudomonas protegens*, while the other one appears to be analogous to the HTH-type transcriptional regulator YidP of *E. coli*, whose function is unknown. The strongest upregulation (almost tenfold) among all transcription factors concerns another HTH member, most likely AnsR. This is the repressor of the *ans* operon, which encodes the enzymes L-asparaginase and L-aspartase [62]. The upregulation is unique to 8-1 and may constitute a possible compensatory mechanism to increase the aspartate levels. The strongest downregulation is that of Spx transcription factor and, while not unique to 8-1, it is more than five times stronger in this strain than in Ym1 (20.62- vs. 3.8-fold). The Spx transcription factors usually interact directly with the RNA polymerase and are mostly involved in oxidative stress; several species of LAB have been reported to contain from two to seven Spx paralogs [63].

We also observed a striking effect regarding the LacI family. Two members were 4.76- and 3.73-fold downregulated in 8-1, the central catabolite regulator CcpA in several genera of LAB [64,65] and a repressor of the maltose operon, respectively. Another member from the same family, the evolved β-galactosidase operon repressor EbgR, was 2.51-fold upregulated in Ym1. This may be related to one of the strongest downregulations of all: an IS5-like transposase was more than a hundredfold downregulated in Ym1, but showed no difference in 8-1 (Supplementary Figure S3). Transposases are reportedly the most abundant genes in nature [66], and some members from the I5 family have been associated with acquired antibiotic/xenobiotic resistance [67]. Of more relevance to the present study, transposable elements have been shown to affect *ebgR* at a much higher rate in response to carbon starvation in *E. coli*. Other β-glucoside operons, such as *bgl*, *cel*, and *asc*, are also known to be regulated by transposons [68]. However, the role of mobile genetic elements in butanol stress remains a largely unexplored area.

### 3.12. Electron Transport

Aerobic respiration in LAB has been known since the 1970s, but is limited to very few species, most notably *Lc. lactis*, and even in them, it occurs only under special conditions [69]. *L. plantarum* does not respire, except when heme and menaquinone are added to the medium [70]. During butanol stress, two possible electron carriers were upregulated: cytochrome oxidase (2.35-fold in 8-1) and FAD-dependent NADH dehydrogenase (2.76-fold in Ym1). Significant upregulation (7.72-fold) of a flavoprotein in Ym1 suggests its action as a fumarate reductase. Fumarate is often used as a terminal electron acceptor in anaerobic eukaryotes as well as in many bacteria [71]. Further studies are needed to elucidate the electron donors for this fumarate reductase in Ym1, as well as the composition of the electron transport chains and their role under butanol stress in both strains.

## 4. Conclusions

The present study is the first to employ transcriptomics in considering the effect of butanol stress on *L. plantarum*. Two strains of the species displaying quite different ability to tolerate butanol, one highly resistant (Ym1) and the second sensitive (8-1), were analysed for their differential gene expression during butanol challenge. DEGs’ analysis revealed the following: (*i*) several species-specific genetic determinants of the butanol stress response, and (*ii*) some of the strain-specific mechanisms responsible for the greater butanol resistance of Ym1. Thus, the results revealing the differential gene expression of Ym1 could be considered as a model scheme for adequate and successful cellular response to butanol stress.

The effects common to both strains are the overexpression of genes encoding SMR, MSF, MMSF, and the toxin-antitoxin system, which provide active transport of toxic metabolites out of the cell and act similarly to efflux pumps in Gram-negative bacteria. Another common effect is the severe downregulation of currently unnecessary and energy-consuming biosynthetic pathways, such as those for nucleotides and tryptophan biosynthesis. The majority of other effects, however, are strain-specific. Uniquely overexpressed in Ym1 chaperone GroES, chaperonin GroEL, and foldase PrsA confirmed their importance for the processes of proper folding and repair of proteins under stress. The suppression of glycerol conversion, causing disturbed glycerophospholipid synthesis in 8-1, reveals another important negative effect of butanol stress. Another fundamental reason for the low butanol tolerance of 8-1 is the strain’s inability to reorganize sugar transport through the cell membrane, as shown by the downregulation of several genes responsible for the PTS transport system, and their insufficient energy supply. Another important difference concerns GntR, a master regulator of cellular processes under stress conditions. Its threefold overexpression in Ym1 (compared with sevenfold downregulation in 8-1) seems to be crucial for the overall fitness of the strain during butanol challenge, regardless of the compensatory expression of other regulatory proteins and transcription factors in both strains. An approach that is well applied by the tolerant Ym1, but does not work in the sensitive 8-1, is the redistribution of aspartate, a metabolite with putative importance for butanol tolerance. A cascade of downregulated enzymes stops the aspartate-consuming biosynthesis of purine and pyrimidine nucleotide, while the TCA-aspartate shuttle we propose here leads to increased aspartate production from citrate and oxaloacetate. Another metabolic reprogramming of TCA cycle intermediates specific for Ym1 leads to overproduction of PEP, which is necessary not only for the carbohydrate transport, but also for the formation of EPS, as suggested by the unique overexpression in Ym1 of genes encoding five different proteins related to EPS biosynthesis. Some of the phenomena observed in *L. plantarum* suggest species-inherited tolerance to butanol. However, in different strains within the species, the butanol tolerance varies considerably and may have a decisive effect on survival.

In conclusion, the determination of genetic factors for butanol tolerance and the mechanisms of cellular response of tolerant strains can serve (*i*) to construct a modified strain with increased tolerance, which can be used as a platform for the production of butanol; and (*ii*) as an opportunity for genetic improvement of butanol tolerance of existing producers. Thus, transcriptomic research in *L. plantarum* would lead to the development of improved butanol producers through new and hitherto unexplored approaches.

## Figures and Tables

**Table 1:** Differentially expressed genes (DEGs) of *L. plantarum* strains under butanol stress that were regulated in opposite directions. The strain Ym1 is highly resistant to butanol, whereas the strain 8-1 is moderately sensitive.

Gene Expression	Gene	Protein	FC */Ym1	FC */8-1
Ym1—upregulated 8-1—downregulated	lp_0230	PTS mannitol transporter subunit IICBA	+6.48	−3.22
	lp_1107, *citD*	Citrate lyase acyl carrier protein	+3.30	−2.96
	lp_1109, *citF*	Citrate (pro-3S)-lyase subunit α	+3.23	−2.12
	lp_1108, *citE*	Citrate (pro-3S)-lyase subunit β	+3.24	−2.19
	lp_0435	GntR family transcriptional regulator	+2.83	−7.82
	lp_3313, *pflB*	Formate C-acetyltransferase	+2.45	−7.41
	lp_3054	NAD(P)-dependent alcohol DH	+2.25	−2.09
	lp_3316	Metal-sulfur cluster assembly factor	+2.25	−2.29
	lp_3487, *galM3*	Galactose mutarotase	+2.21	−2.19
Ym1—downregulated 8-1—upregulated	lp_2037, *aroF*	Chorismate synthase	−2.04	+2.61
	lp_2035, *aroE*	3-P-shikimate 1-carboxyvinyltransferase	−2.27	+2.52
	lp_2113	Iron-sulfur cluster biosynthesis protein	−2.36	+2.17
	lp_1992	Hypothetical protein	−2.02	+2.09
	lp_2038	MFS transporter	−2.17	+2.42
	lp_1955	ABC transporter permease subunit	−3.19	+5.81

* The difference in gene expression was estimated as the fold change (FC) of mRNA encoding the respective protein after cultivation of the strain at 2% (*v*/*v*) butanol challenge for 8 h, compared with those of control cultures grown in MRS. DH, dehydrogenase; PTS, phosphotransferase system.

**Table 2:** Differentially expressed genes (DEGs) encoding PTS transporters in *L. plantarum* strains under butanol stress.

Gene	Name	FC * in Ym1	FC * in 8-1
lp_0230, *pts2CB*	PTS mannitol transporter subunit IICBA	+6.48	−3.22
lp_2097, *fruA*	PTS transporter subunit EIIA	+4.35	+2.48
lp_3654, *pts38C*	PTS sorbitol transporter subunit IIC	+2.29	NC
lp_0286, *pts6C*	PTS cellobiose transporter subunit IIC	+2.55	NC
lp_2531, *pts18CBA*	PTS transporter subunit EIIC	NC	+3.22
lp_0886, *pts11BC*	PTS transporter subunit EIIC	NC	−3.91
lp_3240, *pts28ABC*	PTS transporter subunit EIIC	NC	−2.19
lp_0436, *pts7C*	PTS sugar transporter subunit IIC	NC	−17.13
lp_3507, *pts29C*	PTS sugar transporter subunit IIC	NC	−6.13
lp_3010, *pts23C*	PTS sugar transporter subunit IIC	NC	−3.72
lp_2954	PTS sugar transporter subunit IIC	NC	+2.29
lp_2781, *pts20B*	PTS sugar transporter subunit IIB	NC	−5.44
lp_3653, *pts38BC*	PTS glucitol/sorbitol transporter subunit IIB	NC	−5.28
lp_0232, *pts2A*	PTS sugar transporter subunit IIA	NC	−11.82
lp_2780, *pts20A*	PTS lactose/cellobiose transporter subunit IIA	NC	−11.81
lp_3652, *pts38A*	PTS glucitol/sorbitol transporter subunit IIA	NC	−3.11
lp_0884, *pts11A*	PTS glucose transporter subunit IIA	NC	−2.30
lp_0575, *pts9AB*	PTS mannose transporter subunit IIAB	NC	−6.36
lp_0185, *pts1BCA*	PTS β-glucoside transporter subunit IIBCA	NC	−4.96
lp_2649, *pts19C*	PTS N-acetylgalactosamine transporter IIC	−12.45	−2.88
lp_3009, *pts23B*	PTS sugar transporter subunit IIB	−2.19	−3.02
lp_0265, *pts5ABC*	PTS transporter subunit EIIC	−18.57	−14.69
lp_0264, *pts4ABC*	PTS transporter subunit EIIC	−38.68	−25.45
lp_0170, *dak3*	PTS-dependent dihydroxyacetone kinase phosphotransferase subunit DhaM	−4.86	−164.91

* The difference in gene expression was estimated as the fold change (FC) of mRNA encoding the respective protein after cultivation of the strain at 2% (*v*/*v*) butanol challenge for 8 h, compared with control cultures grown in MRS. NC = no change.

**Table 3:** Differentially expressed genes (DEGs) connected with tryptophan biosynthesis in *L. plantarum* strains under butanol stress.

Gene	Protein	FC * in Ym1	FC * in 8-1
lp_2033, *aroI*	Shikimate kinase	−4.43	NC
lp_2037, *aroF*	Chorismate synthase	−2.04	+2.61
lp_1652, *trpE*	Anthranilate synthase component I	−2.31	NC
lp_1653, *trpG*	Anthranilate synthase component II	−3.25	−2.07
lp_1654, *trpD*	Anthranilate phosphoribosyltransferase	−2.49	NC
lp_1656, *trpF*	Phosphoribosylanthranilate isomerase	−2.33	NC
lp_1658, *trpB*	Tryptophan synthase subunit α	−2.26	NC
lp_1657, *trpA*	Tryptophan synthase subunit β	−2.11	NC

* The difference in gene expression was estimated as the fold change (FC) of mRNA encoding the respective protein after cultivation of the strain at 2% (*v*/*v*) butanol challenge for 8 h, compared with control cultures grown in MRS. NC = no change.

**Table 4:** Differentially expressed genes (DEGs) encoding transcriptional regulators in *L. plantarum* strains under butanol stress.

Gene	Protein	FC * in Ym1	FC * in 8-1
lp_0435	GntR family transcriptional regulator	+2.82	−7.82
lp_2782	GntR family transcriptional regulator	−3.65	NC
lp_3506	GntR family transcriptional regulator	−3.53	NC
lp_0917	Helix-turn-helix transcriptional regulator	NC	+9.96
lp_3290	Helix-turn-helix transcriptional regulator	NC	+2.97
lp_1557	Helix-turn-helix transcriptional regulator	NC	+2.40
lp_0921	Helix-turn-helix transcriptional regulator	NC	+2.06
lp_2782	Helix-turn-helix transcriptional regulator	NC	−3.65
lp_0949	Helix-turn-helix transcriptional regulator	NC	−2.04
lp_3415	Helix-turn-helix transcriptional regulator	+2.88	+2.32
lp_3143	Helix-turn-helix transcriptional regulator	+2.79	NC
lp_2228, *spx3*	Transcriptional regulator Spx	NC	+2.35
lp_3579, *spx5*	Transcriptional regulator Spx	−3.80	−20.62
lp_3479, *galR*	LacI family transcriptional regulator	+2.51	NC
lp_0173	LacI family transcriptional regulator	NC	−4.76
lp_0172	LacI family transcriptional regulator	NC	−3.73
lp_0166, *dak1A*	DhaKLM operon coactivator DhaQ	NC	−3.11
lp_3216	PadR family transcriptional regulator	NC	+4.99
lp_0154	PadR family transcriptional regulator	NC	−2.86
lp_2964	HxlR family transcriptional regulator	NC	+2.90
lp_2804	LysR family transcriptional regulator	+2.62	+2.32
lp_1685	LysR family transcriptional regulator	NC	−3.15
lp_1000	LytR family transcriptional regulator	NC	−2.09
lp_1565	LytTR family transcriptional regulator	−2.39	NC
lp_0274	TetR/AcrR family transcriptional regulator	+2.67	+2.19
lp_1442	Crp/Fnr family transcriptional regulator	+2.05	+2.58
lp_3444	Crp/Fnr family transcriptional regulator	NC	+2.54
lp_2095, *fruR*	DeoR/GlpR transcriptional regulator	+4.02	+3.47
lp_1092, *ttdR*	AraC family transcriptional regulator	−2.60	−2.39
lp_1782, *pyrR2*	PyrR transcriptional regulator	−2.10	NC
lp_2704, *pyrR1*	PyrR transcriptional regulator	−7.10	−3.27
lp_2967	MarR family transcriptional regulator	−2.27	NC
lp_0128	MarR family transcriptional regulator	NC	+2.13
lp_2800b	MarR family transcriptional regulator	NC	−2.35
lp_3113	MerR family transcriptional regulator	+3.06	+3.16
lp_0984	MerR family transcriptional regulator	+2.80	+4.88
lp_2567	MerR family transcriptional regulator	+2.20	+2.31
lp_2854	MerR family transcriptional regulator	NC	+4.14
lp_0281	MerR family transcriptional regulator	NC	+2.50
lp_3013	MerR family transcriptional regulator	NC	+2.43
lp_0992	MerR family transcriptional regulator	NC	+2.07
lp_2063, *lexA*	Transcriptional repressor LexA	−2.31	NC
lp_2708, *pucR*	PucR family transcriptional regulator	−2.43	NC
lp_3508	Response regulator transcription factor	NC	−5.63

* The difference in gene expression was estimated as the fold change (FC) of mRNA encoding the respective protein after cultivation of the strain at 2% (*v*/*v*) butanol challenge for 8 h, compared with control cultures grown in MRS. NC = no change.

## Data Availability

The transcriptomic data have been deposited at GenBank of NCBI (https://www.ncbi.nlm.nih.gov/bioproject/PRJNA675300), under the accession number PRJNA675300.

## Supplementary Materials

The following are available online at https://www.mdpi.com/2073-4425/12/2/181/s1, Table S1: *L. plantarum* whole transcriptome sequencing—raw data and trimming data stats; Figure S1: Boxplot of expression difference between samples. Boxplots show the corresponding sample’s expression distribution based on a percentile (median, 50%, 75%, maximum, and minimum) based on raw signal (read count), Log2 transformation of reading count+1, and TMM Normalization; Figure S2: Transcriptome analysis of *L. plantarum* strains Ym1 and 8-1 under conditions of butanol stress. Up- and downregulated count by fold change and *p*-value. Figure S3: Heat maps demonstrating DEGs with the largest deviations in fold change (FC) of the gene expression during butanol stress of *L. plantarum* strains Ym1 (a) and 8-1 (b).
