# Supplementary material for: Butanol Tolerance of Lactiplantibacillus plantarum: A Transcriptome Study
Source: Genes (Basel). 2021 Jan 27;12(2):181. doi: 10.3390/genes12020181 (PMC7911632; doi:10.3390/genes12020181)
Supplement: Supplementary file 1 [file genes-12-00181-s001.pdf]

Supplementary Materials

Table S1. *Lactiplantibacillus plantarum* whole transcriptome sequencing - raw data and trimming data stats.

Index	Sample ID	Total read bases*	Total reads	GC (%)	Q20 (%)	Q30 (%)
Raw data						
1	8-1-2	4,898,324,462	48,498,262	45.62	98.67	95.82
2	8-1-control	4,648,502,578	46,024,778	45.34	98.74	95.94
3	Ym1-2	5,348,720,630	52,957,630	46.00	98.56	95.42
4	Ym1-control	4,802,150,848	47,546,048	45.60	98.70	95.73
Trimming data						
1	8-1-2	4,809,635,855	47,850,304	45.63	99.04	96.35
2	8-1-control	4,571,351,118	45,493,304	45.35	99.06	96.41
3	Ym1-2	5,231,546,807	52,346,352	45.98	98.90	95.93
4	Ym1-control	4,707,100,581	47,063,250	45.58	98.99	96.16

*Legend: Total read bases = Total reads x Read length; Total read bases is the total number of bases sequenced; Total reads is the total number of reads; GC (%) is the molar ratio of (G+C); Q20 (%): Ratio of bases that have PHRED quality score greater than or equal to 20; Q30 (%): Ratio of bases that have PHRED quality score greater than or equal to 30.

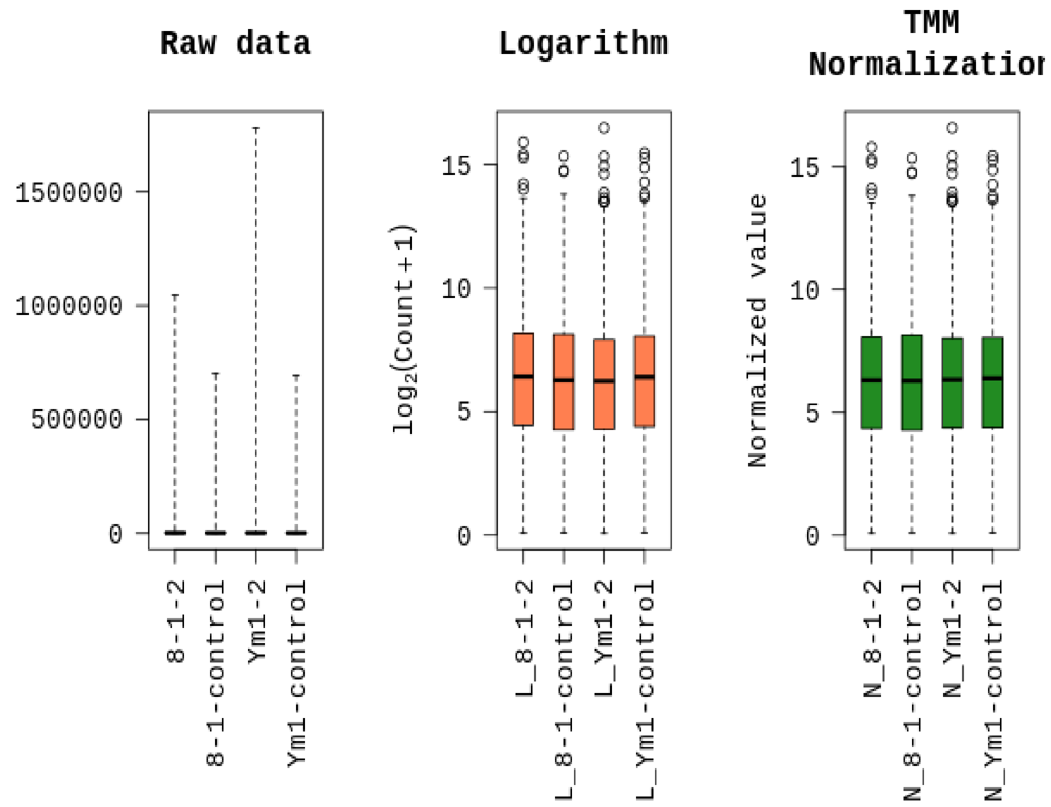

Figure S1. Boxplot of Expression Difference between samples. Boxplots show the corresponding sample's expression distribution based on percentile (median, 50 percentile, 75 percentile, maximum and minimum) based on raw signal (read count), Log2 transformation of read count+1 and TMM Normalization.

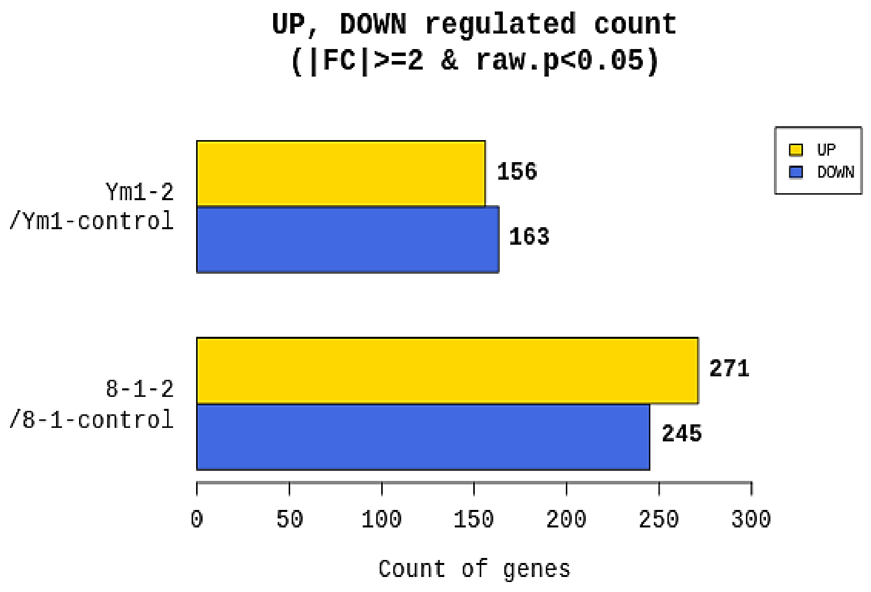

Fig. S2. Transcriptome analysis of *L. plantarum* strains Ym1 and 8-1 under conditions of butanol stress. Up, Down Regulated Count by Fold Change.

Figure S3. Heat maps demonstrating DEGs with the largest deviations in fold change (FC) of the gene expression during butanol stress of *L. plantarum* strains Ym1 (a) and 8-1 (b). Legend:

-30 -20 -10 -5 ±2 5 10 20 30

(a)

Protein_ID	Product	FC/Ym1	FC/8-1
WP_003641313.1	Class II Fumarate Hydratase		
WP_011102288.1	Wxl Domain-Containing Protein		
WP_003641854.1	Hypothetical Protein		
WP_011100948.1	Hypothetical Protein		
WP_003641618.1	Cell Surface Protein		
WP_050577163.1	DUF916 And DUF3324 Domain-Containing Protein		
WP_011102286.1	Cell Surface Protein		
WP_011101260.1	Flavocytochrome C		
WP_003644108.1	L-Lactate Dehydrogenase		
	tRNA-Ser		
WP_011100926.1	PTS Mannitol Transporter Subunit IICBA		
WP_003643605.1	Bifunctional Acetaldehyde-Coa/Alcohol Dehydrogenase		
WP_003642522.1	Aldo/Keto Reductase		
WP_003645041.1	Alcohol Dehydrogenase Catalytic Domain-Containing Protein		
WP_011102053.1	FAD-Dependent Oxidoreductase		
WP_011101921.1	Aldo/Keto Reductase		
WP_011101561.1	2-C-Methyl-D-Erythritol 4-Phosphate Cytidylyltransferase		
WP_003646028.1	Phosphoenolpyruvate Synthase		
WP_011101394.1	Lyse Family Transporter		
WP_011102123.1	GNAT Family N-Acetyltransferase		
WP_011102074.1	Foldase		
WP_003641299.1	Anion Permease		
WP_011102185.1	Pyridoxamine 5'-Phosphate Oxidase Family Protein		
WP_011101650.1	PTS Transporter Subunit EIIA		
WP_003644568.1	1-Phosphofructokinase		
WP_003641765.1	PTS-Dependent Dihydroxyacetone Kinase Phosphotransferase Subunit Dham		
WP_003642544.1	6-Phospho-Beta-Glucosidase		
WP_011101228.1	Hypothetical Protein		
WP_011101884.1	DMT Family Transporter		
WP_003642546.1	ABC Transporter ATP-Binding Protein		
WP_011102035.1	Asparagine Synthase (Glutamine-Hydrolyzing)		
WP_011102021.1	Transglycosylase		

WP_003643977.1	ABC Transporter Permease Subunit
WP_011101671.1	Branched-Chain Alpha-Keto Acid Dehydrogenase Subunit E2
WP_011101161.1	Amino Acid ABC Transporter ATP-Binding Protein
WP_003645874.1	Purine Permease
WP_003640822.1	Dihydrolipoyl Dehydrogenase
WP_003639428.1	Bifunctional Pyr Operon Transcriptional Regulator/Uracil Phosphoribosyltransferase Pyrr
WP_003641766.1	Aquaporin Family Protein
WP_003643774.1	Type 1 Glycerol-3-Phosphate Oxidase
WP_003641199.1	Hypothetical Protein
WP_003643557.1	Branched-Chain Amino Acid Transport System II Carrier Protein
WP_003641437.1	Uracil Transporter
WP_021731937.1	Aquaporin Family Protein
WP_011101866.1	PTS N-Acetylgalactosamine Transporter Subunit IIC
WP_164920167.1	PTS Transporter Subunit EIIC
WP_003643703.1	Trehalose Operon Repressor
WP_011100943.1	Alpha,Alpha-Phosphotrehalase
WP_011101885.1	Orotate Phosphoribosyltransferase
WP_003642610.1	Orotidine-5'-Phosphate Decarboxylase
WP_003645881.1	Dihydroorotate Dehydrogenase
WP_011101886.1	Carbamoyl-Phosphate Synthase Large Subunit
WP_003643705.1	PTS Transporter Subunit EIIC
WP_011101887.1	Carbamoyl Phosphate Synthase Small Subunit
WP_003644720.1	Dihydroorotase
WP_011101888.1	Aspartate Carbamoyltransferase Catalytic Subunit
WP_076612162.1	IS5-Like Element Islpl3 Family Transposase

(b)

Protein_ID	Product	FC/8-1	FC/Ym1
WP_011100898.1	Hsp20/Alpha Crystallin Family Protein		
WP_011102037.1	NAD(P)H-Binding Protein		
WP_011102123.1	GNAT Family N-Acetyltransferase		
WP_003643361.1	Lysm Peptidoglycan-Binding Domain-Containing Protein		
WP_003640551.1	Hypothetical Protein		
WP_011102082.1	Alpha-Glucosidase		
WP_003642122.1	Lysm Peptidoglycan-Binding Domain-Containing Protein		
WP_011102288.1	Wxl Domain-Containing Protein		
WP_003643721.1	ABC Transporter Permease		
WP_011101381.1	SLC13 Family Permease		
WP_011101394.1	Lyse Family Transporter		
WP_011101561.1	2-C-Methyl-D-Erythritol 4-Phosphate Cytidylyltransferase		
WP_011101992.1	Wxl Domain-Containing Protein		
WP_003641618.1	Cell Surface Protein		
WP_003641873.1	ABC Transporter ATP-Binding Protein		
WP_050577163.1	DUF916 And DUF3324 Domain-Containing Protein		
WP_011102286.1	Cell Surface Protein		
WP_003641165.1	Helix-Turn-Helix Transcriptional Regulator		
WP_003643508.1	Hsp20/Alpha Crystallin Family Protein		
WP_011100957.1	Lysm Peptidoglycan-Binding Domain-Containing Protein		
WP_003644885.1	SDR Family Oxidoreductase		
.	Trna-Lys		
WP_003639252.1	Hypothetical Protein		
WP_003587210.1	DNA Starvation/Stationary Phase Protection Protein		
WP_011100859.1	Nitroreductase		
WP_011101379.1	Sulfate Adenylyltransferase		
WP_003645852.1	Hypothetical Protein		
WP_011101378.1	Sulfotransferase Family Protein		
WP_011102185.1	Pyridoxamine 5'-Phosphate Oxidase Family Protein		
WP_011101384.1	Hypothetical Protein		
WP_003641846.1	Hypothetical Protein		
	tRNA-Thr		
WP_003642504.1	Multidrug Efflux SMR Transporter		
WP_011101730.1	Yxea Family Protein		
WP_003645041.1	Alcohol Dehydrogenase Catalytic Domain-Containing Protein		
WP_003642309.1	Cupredoxin Domain-Containing Protein		
WP_011102022.1	Copper-Translocating P-Type Atpase		
WP_003644468.1	ABC Transporter Permease Subunit		

	tRNA-Thr
WP_011101947.1	Hypothetical Protein
WP_011101407.1	6,7-Dimethyl-8-Ribityllumazine Synthase
WP_011101990.1	LPXTG Cell Wall Anchor Domain-Containing Protein
WP_003643453.1	PadR Family Transcriptional Regulator
WP_003643301.1	Universal Stress Protein
WP_011100969.1	Hypothetical Protein
WP_003641218.1	MerR Family Transcriptional Regulator
WP_003640792.1	Hypothetical Protein
WP_060684211.1	Pyruvate Oxidase
WP_003641796.1	3-Phosphoserine/Phosphohydroxythreonine Transaminase
WP_011102065.1	ROK Family Protein
WP_003641026.1	M15 Family Metallopeptidase
WP_003645874.1	Purine Permease
WP_003642982.1	DUF1836 Domain-Containing Protein
WP_003641713.1	Larc Family Nickel Insertion Protein
WP_003643083.1	Alpha/Beta Fold Hydrolase
WP_033098941.1	Lysm Peptidoglycan-Binding Domain-Containing Protein
WP_011101502.1	Acetyl-Coa Carboxylase Biotin Carboxylase Subunit
WP_003643084.1	Gntr Family Transcriptional Regulator
WP_003643223.1	Gluconate Permease
WP_003640947.1	Cell Surface Protein
WP_003641746.1	Energy-Coupling Factor Transporter Transmembrane Protein Ecft
WP_003642541.1	GntR Family Transcriptional Regulator
WP_003643055.1	Helix-Turn-Helix Transcriptional Regulator
WP_003643656.1	Aquaporin Family Protein
WP_011101411.1	Wxl Domain-Containing Protein
WP_011102234.1	Accessory Gene Regulator Agrb
WP_011102035.1	Asparagine Synthase (Glutamine-Hydrolyzing)
WP_011102003.1	PTS Sugar Transporter Subunit IIC
WP_003643677.1	Laci Family Transcriptional Regulator
WP_011101161.1	Amino Acid ABC Transporter ATP-Binding Protein
WP_003644020.1	PTS Transporter Subunit EIIC
WP_015640940.1	Hypothetical Protein
WP_003640790.1	Amino Acid ABC Transporter ATP-Binding Protein
WP_003642044.1	Serine Transporter
WP_003644021.1	Chbg/Hpnk Family Deacetylase
WP_003643082.1	Hypothetical Protein
WP_011101904.1	Alpha-Glycosidase
WP_003642507.1	Cation Transporter
WP_003642097.1	Yuei Family Protein

WP_003642526.1	Amino Acid Permease
WP_003641437.1	Uracil Transporter
WP_003641768.1	LacI Family Transcriptional Regulator
WP_003643068.1	Glyceraldehyde 3-Phosphate Reductase
WP_003641781.1	PTS Beta-Glucoside Transporter IIBCA
WP_003641776.1	Glycoside Hydrolase Family 65 Protein
WP_003641787.1	Alpha-Glucosidase
WP_011102232.1	GHKL Domain-Containing Protein
WP_011100909.1	Extracellular Solute-Binding Protein
WP_003640791.1	ABC Transporter Substrate-Binding Protein/Permease
WP_003641597.1	PTS Glucitol/Sorbitol Transporter IIB
WP_003641212.1	MFS Transporter
WP_003645881.1	Dihydroorotate Dehydrogenase
WP_003639490.1	PTS Sugar Transporter Subunit IIB
WP_003642610.1	Orotidine-5'-Phosphate Decarboxylase
WP_003641775.1	ABC Transporter ATP-Binding Protein
WP_003641774.1	Alpha-Amylase
WP_011102231.1	Response Regulator Transcription Factor
WP_011101324.1	Prepilin-Type N-Terminal
WP_003641549.1	Cleavage/Methylation Domain Protein
WP_011102193.1	N-Acetylmuramic Acid 6-P- Etherase
WP_003641771.1	Sugar ABC Transporter Permease
WP_011100883.1	Nickel-Dependent Lactate Racemase
WP_011102021.1	Transglycosylase
WP_011100884.1	Nickel Pincer Cofactor Biosynthesis Protein LarB
WP_011101885.1	Orotate Phosphoribosyltransferase
WP_003643568.1	PTS Sugar Transporter Subunit IIC
WP_011102182.1	Beta-Galactosidase
WP_003643005.1	Phosphoenolpyruvate Carboxykinase (ATP)
WP_011101057.1	PTS Mannose Transporter Subunit IIAB
WP_011100910.1	Sugar ABC Transporter
WP_003641772.1	Sugar ABC Transporter Permease
WP_003641704.1	Hypothetical Protein
WP_003640464.1	Glycoside Hydrolase Family 65 Protein
WP_011101908.1	GNAT Family N-Acetyltransferase
WP_013356041.1	N-Acetylglucosamine Kinase
WP_011101886.1	Carbamoyl-Phosphate Synthase Large Subunit
WP_003642836.1	Formate C-Acetyltransferase
WP_003641769.1	Alpha-Glucosidase
WP_003642552.1	DUF871 Domain-Containing Protein
WP_003642098.1	Hypothetical Protein
WP_003643729.1	Acetate Kinase
WP_011101005.1	GntR Family Transcriptional Regulator
WP_011101888.1	Asp Carbamoyltransferase Catalytic Subunit

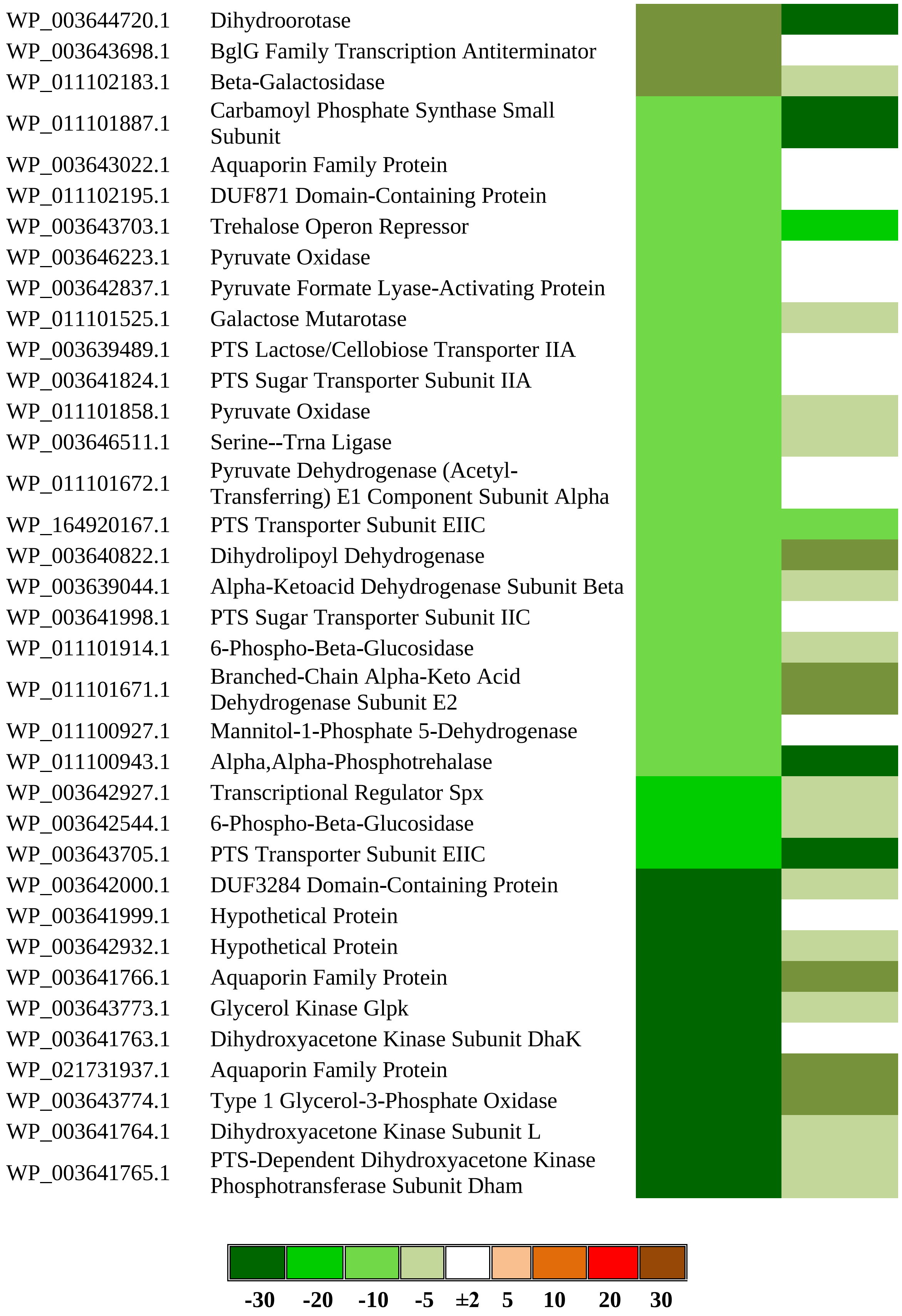
WP_003644720.1 Dihydroorotase
WP_003643698.1 BglG Family Transcription Antiterminator
WP_011102183.1 Beta-Galactosidase
WP_011101887.1 Carbamoyl Phosphate Synthase Small Subunit
WP_003643022.1 Aquaporin Family Protein
WP_011102195.1 DUF871 Domain-Containing Protein
WP_003643703.1 Trehalose Operon Repressor
WP_003646223.1 Pyruvate Oxidase
WP_003642837.1 Pyruvate Formate Lyase-Activating Protein
WP_011101525.1 Galactose Mutarotase
WP_003639489.1 PTS Lactose/Cellobiose Transporter IIA
WP_003641824.1 PTS Sugar Transporter Subunit IIA
WP_011101858.1 Pyruvate Oxidase
WP_003646511.1 Serine--Trna Ligase
WP_011101672.1 Pyruvate Dehydrogenase (Acetyl-Transferring) E1 Component Subunit Alpha
WP_164920167.1 PTS Transporter Subunit EIIC
WP_003640822.1 Dihydrolipoyl Dehydrogenase
WP_003639044.1 Alpha-Ketoacid Dehydrogenase Subunit Beta
WP_003641998.1 PTS Sugar Transporter Subunit IIC
WP_011101914.1 6-Phospho-Beta-Glucosidase
WP_011101671.1 Branched-Chain Alpha-Keto Acid Dehydrogenase Subunit E2
WP_011100927.1 Mannitol-1-Phosphate 5-Dehydrogenase
WP_011100943.1 Alpha,Alpha-Phosphotrehalase
WP_003642927.1 Transcriptional Regulator Spx
WP_003642544.1 6-Phospho-Beta-Glucosidase
WP_003643705.1 PTS Transporter Subunit EIIC
WP_003642000.1 DUF3284 Domain-Containing Protein
WP_003641999.1 Hypothetical Protein
WP_003642932.1 Hypothetical Protein
WP_003641766.1 Aquaporin Family Protein
WP_003643773.1 Glycerol Kinase Glpk
WP_003641763.1 Dihydroxyacetone Kinase Subunit DhaK
WP_021731937.1 Aquaporin Family Protein
WP_003643774.1 Type 1 Glycerol-3-Phosphate Oxidase
WP_003641764.1 Dihydroxyacetone Kinase Subunit L
WP_003641765.1 PTS-Dependent Dihydroxyacetone Kinase Phosphotransferase Subunit Dham
-30 -20 -10 -5 ±2 5 10 20 30
